# Shorter Men Live Longer: Association of Height with Longevity and *FOXO3* Genotype in American Men of Japanese Ancestry

**DOI:** 10.1371/journal.pone.0094385

**Published:** 2014-05-07

**Authors:** Qimei He, Brian J. Morris, John S. Grove, Helen Petrovitch, Webster Ross, Kamal H. Masaki, Beatriz Rodriguez, Randi Chen, Timothy A. Donlon, D. Craig Willcox, Bradley J. Willcox

**Affiliations:** 1 Honolulu Heart Program/Honolulu-Asia Aging Study, Physicians’ Office Tower, Kuakini Medical Center, Honolulu, Hawaii, United States of America; 2 Pacific Health Research and Education Institute of the Veterans Affairs Pacific Islands Health Care System, Honolulu, Hawaii; 3 Department of Geriatric Medicine, John A. Burns School of Medicine, University of Hawaii, Honolulu, Hawaii, United States of America; 4 School of Medical Sciences, University of Sydney, Sydney, New South Wales, Australia; 5 Department of Public Health, University of Hawaii at Manoa, Honolulu, Hawaii; 6 Instituto Tecnologico de Monterrey, Monterrey, Mexico; 7 Department of Research, Kuakini Medical Center, Honolulu, Hawaii, United States of America; 8 Department of Human Welfare, Okinawa University, Ginowan, Okinawa, Japan; “Mario Negri” Institute for Pharmacological Research, Italy

## Abstract

**Objectives:**

To determine the relation between height, *FOXO3* genotype and age of death in humans.

**Methods:**

Observational study of 8,003 American men of Japanese ancestry from the Honolulu Heart Program/Honolulu-Asia Aging Study (HHP/HAAS), a genetically and culturally homogeneous cohort followed for over 40 years. A Cox regression model with age as the time scale, stratified by year of birth, was used to estimate the effect of baseline height on mortality during follow-up. An analysis of height and longevity-associated variants of the key regulatory gene in the insulin/IGF-1 signaling (IIS) pathway, *FOXO3*, was performed in a HHP-HAAS subpopulation. A study of fasting insulin level and height was conducted in another HHP-HAAS subpopulation.

**Results:**

A positive association was found between baseline height and all-cause mortality (RR = 1.007; 95% CI 1.003–1.011; *P* = 0.002) over the follow-up period. Adjustments for possible confounding variables reduced this association only slightly (RR = 1.006; 95% CI 1.002–1.010; *P* = 0.007). In addition, height was positively associated with all cancer mortality and mortality from cancer unrelated to smoking. A Cox regression model with time-dependent covariates showed that relative risk for baseline height on mortality increased as the population aged. Comparison of genotypes of a longevity-associated single nucleotide polymorphism in *FOXO3* showed that the longevity allele was inversely associated with height. This finding was consistent with prior findings in model organisms of aging. Height was also positively associated with fasting blood insulin level, a risk factor for mortality. Regression analysis of fasting insulin level (mIU/L) on height (cm) adjusting for the age both data were collected yielded a regression coefficient of 0.26 (95% CI 0.10–0.42; *P* = 0.001).

**Conclusion:**

Height in mid-life is positively associated with mortality, with shorter stature predicting longer lifespan. Height was, moreover, associated with fasting insulin level and the longevity genotype of *FOXO3*, consistent with a mechanistic role for the IIS pathway.

## Introduction

Several studies have found an inverse association between height and mortality, especially for deaths arising from respiratory diseases, coronary heart disease (CHD) and stroke [1]–[8]. In contrast, other studies found a direct relationship between height and mortality [9]–[13], one caveat being those infants born underweight who undergo post-partum compensatory growth and have increased risk of cardiovascular disease [14], [15]. The higher caloric intake of taller people may predispose them to increased risk of cancer and thus premature death [2], [10], [16]. In support, a study of 1,915 healthy nonsmoking Japanese American men from the Honolulu Heart Program (HHP), followed for 36 years, found lower mortality in those whose energy consumption was 15% below average [17].

The sex difference in lifespan has been attributed in part to the average shorter stature of women [18]. Reduced insulin/insulin-like growth factor-1 (IGF-1) signaling is associated with increased lifespan and shorter height in women [11]. These associations were most pronounced for genetic variants of the human growth hormone (*GH1*), *IGF1* and insulin receptor substrate-1 (*IRS1*) genes [11].

Studies to date suffer from confounding factors such as population stratification artifact and from racial, ethnic, dietary, socio-economic and other confounders, as well as inadequate length of follow-up. These may be contributing to the lack of consensus about whether height affects lifespan positively or negatively. In the present study, we examined this question using data from the HHP/Honolulu-Asia Aging Study (HAAS), a population-based study of genetically homogeneous Japanese-American men, who have been followed-up for more than 40 years. Given that smaller body size is a phenotype that is strongly associated with increased lifespan in model organisms of aging, and may be mediated through energy-sensing pathways, particularly the insulin/IGF-1 (IIS) signaling pathway, we hypothesized that shorter height in humans would be associated with increased longevity and genotype of *FOXO3*, a key IIS regulatory gene associated with human longevity [19] and reduced insulin signaling. The present study therefore assessed whether height was associated with longevity, *FOXO3* genotype and fasting insulin level, a marker of insulin sensitivity.

## Materials and Methods

### Study population

The study utilized the HHP/HAAS dataset. The HHP is a population-based, prospective cohort comprising 8,006 Japanese-American men who were born between 1900 and 1919 and recruited to study cardiovascular disease. All were residents of the island of Oahu, Hawaii, at the time of study enrolment (1965–1968), and were aged 45 to 68 years (mean 54 years). Approximately 12% were born in Japan and the rest were mainly second generation Japanese-Americans. Approximately 12% of the latter went to live in Japan as children at an average age of 5 years. Most of this subset continued to reside in Japan until age 17–25 years before returning to Oahu. Recruitment, design, subjects and procedures have been described elsewhere [20]–[23]. The HAAS was developed from within the HHP in 1991 for the purpose of studying dementia and conditions related to aging [24]. The HHP/HAAS has a surveillance system to continuously track morbidity from major cardiovascular diseases and to determine the mortality status of participants. Information on incident CHD and stroke, as well as mortality from all causes, were obtained through monitoring obituaries in local newspapers (in both English and Japanese), surveillance of hospital discharge records and state health department records of death certificates. A follow-up survey in the 1991–1993 examination identified mortality information for all but 5 men [25]. The original complete HHP cohort has been re-examined 11 times up until 2012. As of July 2008, 7,063 subjects had a death date recorded in our surveillance system and most of the remaining 943 participants were still alive, with age ranging from 88 to 106 years.

### Data collection: measurement of height and other parameters

HHP participants received a baseline physical examination when recruited in 1965–1968. Standing height (was measured in inches, and then converted into cm in the present analysis) and other demographic and epidemiological variables were recorded. The latter included weight, blood pressure (BP), smoking status, blood sample random serum glucose and uric acid, medical history, alcohol consumption and dietary nutrition intake based on a questionnaire of 24 hour dietary recall, amongst other variables. The procedures used, together with a document for participants explaining the research project and a consent form, were each approved in writing by the Kuakini Medical Center institutional ethical review committee. The study participants read the explanation and then signed the consent form [22].

In the HHP follow-up Exam 4 in 1991–1993 (also known as HAAS Exam 1), 3,741 of the original cohort participated. This reduced number was a consequence of over half of the original cohort having died. The parameters above were re-recorded. Fasting insulin was measured in 3,458 of these. In this examination, standing height was measured (in cm) [23].

### Subjects for *FOXO3* genetic association study

Later (in 2007), a subset of 614 subjects who participated in Exam 4 were chosen for a genetic association study of single nucleotide polymorphisms (SNPs) of *FOXO3* and longevity, as reported previously [19]. We used genotype data for 587 of those subjects for whom we had height data and examined the relationship between the *FOXO3* SNP (rs 2802292) most strongly associated with longevity in our prior study [19] and height.

### Data analysis

Cox’s proportional hazard model was used to analyze the survival data [26] in order to investigate the relation between baseline (mid-life) height and mortality during the follow-up period. For men without mortality information, either because they were still alive or had been lost to follow-up, their age (as censored) at latest contact was used in the model. Due to the rapid change of human mortality rate from middle to later life, as well as a large age range in the study participants, age was used as the time scale in the analysis. We were aware of the possibility of a cohort effect on height due to nutritional status pre-partum and during pediatric development. Therefore, stratification by year of birth was used in the Cox model, which assumes each stratum has its own baseline risk function (or risk curve) and the relative risk (RR) for each of the experimental variables is the same across strata. More specifically, a Cox regression model with age as the time scale and stratified by year of birth was used in all survival analyses for this study.

We also investigated the potential confounding effects of other risk factors on the relation between longevity and baseline height. For alcohol usage, non-drinkers and heavy drinkers (>480 g/month) were compared with moderate drinkers (≤480 g/month). Heavy smokers (>48 pack-year) and moderate smokers (≤48 pack-year) were compared to non-smokers. For other continuous variables, including systolic and diastolic blood pressure, random serum glucose and serum uric acid, quartiles were used in the analysis, and the high quartile and the middle two quartiles were compared to the low quartile. In our analysis, body mass index (BMI) showed a U-shaped relation to mortality. Therefore the comparisons used here are the low quartile vs. the middle two quartiles, and the high quartile vs. the middle two quartiles. Because our analysis showed that the group of participants who had lived in Japan in their early life (either born in Japan, or born in Hawaii and sent back to Japan for a period when they were young) had an advantage in survival compared to those who had never lived in Japan when young, an indicator variable was created for this subgroup of participants, and was included in our analysis as a potential confounder.

Subsequently, we analyzed all possible confounding variables individually in separate models with baseline height and baseline age. Then all variables that showed a significant association with all-cause mortality in the models with baseline height and baseline age only, were adjusted in the final model, to assess the association between height and mortality.

*FOXO3* genotype and fasting insulin are two important variables related to mortality and both are related to insulin signaling. Longevity-associated *FOXO3* genotypes are associated with reduced insulin-signaling, as are lower fasting insulin levels [19]. Therefore, we examined (i) the relation between height and *FOXO3* genotype in the case-control study subset described above and (ii) the relation between fasting insulin level and height, using data from HHP Exam 4. Pearson’s X^2^ test was used for contingency tables analysis and ANOVA was used for analysis of the association of height with genotype.

To assess the relative risk of baseline height in different stages of life, a Cox regression model with time-dependent variables was used to examine the change in relative risk of baseline height on mortality at different life stages. First, index variables were created for different life stages (age ≤70, 71–80, 81–85 and >95 years). These variables were ascribed a value of 1 in each of the corresponding life stage, and 0 outside that life stage. To estimate the relative risk of height at each of these life stages, the products of baseline height and each of the index variables were entered into the Cox regression model together with confounding factors. In this manner, the estimated relative risk of height for each of the life stages could be obtained in one model and the relation between the relative risks of baseline height between different life stages could be assessed. (See Appendix S1 for more detailed information about the models used to compare the relative risks between baseline height at different life stages and mortality.)

## Results

The HHP study began in 1965 and initially identified 12,261 Japanese-American men born between 1900 and 1919 and having a residential address on Oahu. Of these, 8,006 participated and were followed for more than 40 years or until death. Since height data at baseline were not available for 3 subjects, only 8,003 were used in the present analysis.

**Figure 1:**
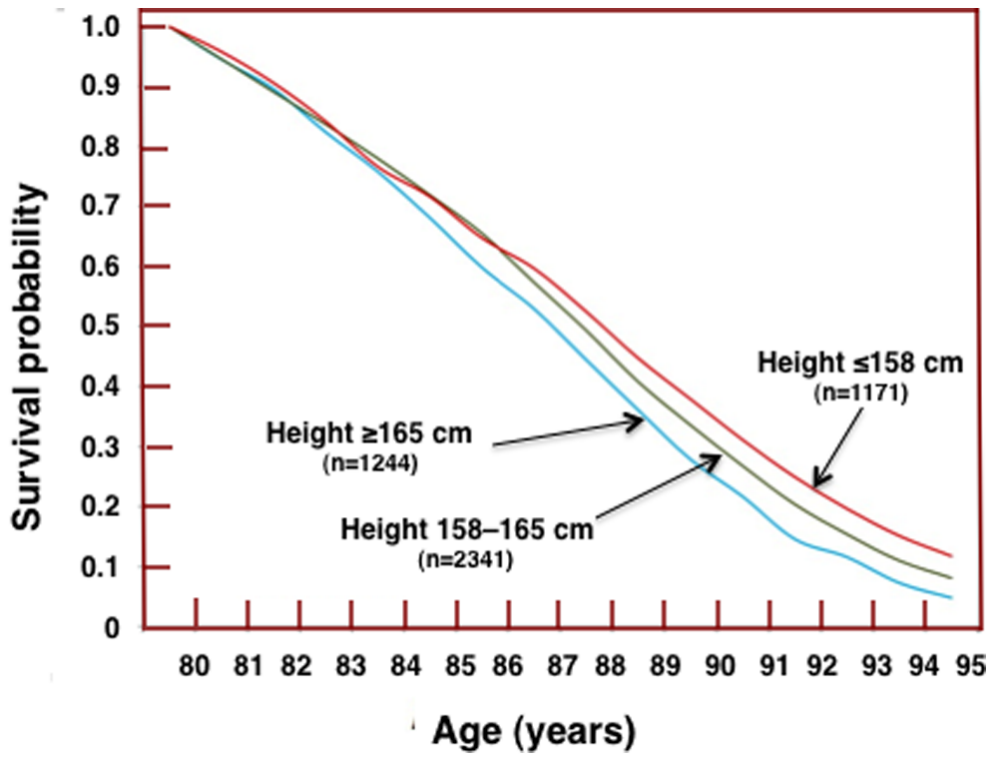
Survival curves for groups differing in baseline height between age 80 and 95 years. Wilcoxon test for difference between the three groups: *P* = 0.0001.

The baseline characteristics of the study population are presented in Table 1. Baseline height by age group is presented in Table 2. Individuals in the oldest age group (65–68 years old) were on average 4.6 cm shorter than those in the youngest age group (45–49 years old). We compared survival curves between participants who were 165 cm or taller in height, those who were 158 cm or shorter, and those whose height was between 158 cm and 165 cm. We found no significant difference between the groups for survival prior to the age of 80 years. Survival curves in the follow-up for these three groups of people differed significantly between age 80 and 95 years (Wilcoxon test for difference: X^2^_2 d.f._ = 17.9; *P* = 0.0001; Figure 1).

**Table 1:** Distribution of demographic and epidemiological variables at baseline.

Variable name	n	Mean ± SD	Range	Low quartile	High quartile
Age at baseline (years)	8006	54.9±5.6	45.8–68.5	50.2	59.2
Height at baseline (cm)	8003	162.8±5.8	140–188	160	168
Systolic BP (mmHg)	8006	134.1±21.0	79.6–264	119.3	146.0
Diastolic BP (mmHg)	8006	82.2±11.6	40.0–172	74.7	89.3
Body mass index (kg/m^2^)	8001	23.8±3.1	14.3–44.6	21.7	25.8
Random serum glucose (mg/dl)	7977	162±58	40–671	122	189
Serum uric acid (mg/dL)	7971	59.9±15.1	7–148	50	69
Alcohol use (g/month)*	7988	388±691	0–10320	0	482
Smoking (pack-year)†	7918	31.6±29.8	0–192	0	48
Major disease at baseline§	8006	1501 (18.7%) with major disease			
Married at baseline	8000	7103 (88.8%) married at baseline			
Lived in Japan when young¶	8006	2288 (28.6%) had lived in Japan			

* 2,985 (37.4%) of subjects were non-drinkers.

† 2,242 (28.3%) had never smoked.

§ CHD, CVD, cancer, diabetes, liver, renal disease, lung disease or other chronic diseases at baseline.

¶ Subjects either born in Japan or born in Oahu and then sent back to Japan, where they lived during early life.

**Table 2:** Height by age group at baseline (Honolulu Heart Program Exam 1).

Age at Exam 1	n	Height (cm) *	Range
46–49	1524	164±5.6	142–188
50–54	2897	164±5.6	145–183
55–59	1688	163±5.8	140–183
60–64	1348	161±5.8	145–180
65–68	546	160±5.3	142–175

*Mean ± SD. Height was measured in inches and converted to cm before analysis.

Using a Cox regression model with age as the time scale, stratified by birth year and adjusting for baseline age only, the RR for mortality associated with every 1 cm increase in baseline height was 1.007 (95% CI 1.003–1.011; *P* = 0.002). When additional confounding risk factors for mortality at baseline (BMI, existing major chronic conditions such as CHD, stroke, cancer, diabetes, liver and renal diseases, chronic obstructive lung diseases and other chronic diseases at baseline, systolic blood pressure, alcohol use, smoking status, serum glucose, cholesterol and uric acid, marital status at baseline and ever lived in Japan) were included in the model, the RR of baseline height for mortality decreased slightly to 1.006 for every 1 cm increase in baseline height (95% CI 1.002–1.010; *P* = 0.007). Table 3 shows results obtained for the final multivariate model.

**Table 3:** Results of multivariate Cox’s proportional hazard model for mortality.

Variable name	Comparison	Relative risk (95% CI)	*P*
Age at baseline*		0.973 (0.947–0.999)	0.042
Height (per cm increase)		1.006 (1.002–1.010)	0.007
Major disease at baseline*	Yes vs. no	1.311 (1.234–1.393)	<0.001
Systolic blood pressure	High vs. low	1.426 (1.328–1.531)	<0.001
	Medium vs. low	1.112 (1.046–1.181)	<0.001
Random serum glucose	High vs. low	1.272 (1.188–1.362)	<0.001
	Medium vs. low	1.034 (0.975–1.097)	0.27
Serum uric acid	High vs. low	1.209 (1.1266–1.297)	<0.001
	Medium vs. low	1.055 (0.995–1.118)	0.07
Alcohol use	None vs. moderate	1.110 (1.050–1.174)	<0.001
	Heavy vs. moderate	1.126 (1.057–1.199)	<0.001
Married at Exam 1	Yes vs. no	0.856 (0.795–0.922)	<0.001
Smoking	Moderate. vs. none	1.309 (1.234–1.388)	<0.001
	Heavy vs. none	1.640 (1.534–1.754)	<0.001
BMI at baseline	Low vs. median	1.126 (1.061–1.195)	<0.001
	High vs. median	1.062 (1.001–1.126)	0.047
Lived in Japan when young	Yes vs. No	0.919 (0.868–0.972)	0.003

*For units of each measurement see Table 1.

Cause-specific mortality data for the HHP/HAAS cohort until the end of 1999 were available and were used to examine the relation between height and cause-specific mortality (Table 4). Cancer mortality, especially non-smoking related cancer, was significantly associated with greater baseline height, while smoking-related cancer was not. CHD and respiratory disease mortality were not significantly associated with baseline height.

**Table 4:** Relative risk for height (per cm increase) for various conditions using data up to the end of 1999.

Type of mortality	No. of events	Age adjusted			RF adjusted for age **		
		RR	95% CI	*P* value	RR	95% CI	*P*
Total mortality	5154	1.006	1.002–1.011	0.010	1.005	1.000–1.010	0.076
All cancer	1584	1.013	1.004–1.022	0.004	1.010	1.001–1.019	0.029
• smoking related*	632	1.008	0.995–1.022	0.238	1.002	0.988–1.017	0.74
• not smoking related	952	1.016	1.005–1.028	0.006	1.015	1.003–1.027	0.012
• stomach (ICD 151)	249	1.009	0.987–1.031	0.434	1.010	0.987–1.033	0.41
• colorectal (ICD 153, 154)	209	1.005	0.981–1.029	0.687	1.003	0.978–1.028	0.82
• prostate (ICD 185)	170	1.025	0.998–1.053	0.072	1.023	0.995–1.051	0.10
• hematopoietic (ICD 200–8)	120	1.038	1.006–1.072	0.019	1.041	1.008–1.075	0.015
CHD	757	1.007	0.994–1.020	0.291	1.003	0.991–1.017	0.60
Respiratory disease	389	0.999	0.981–1.016	0.877	1.000	0.982–1.018	0.99
Stroke	493	0.994	0.979–1.010	0.484	0.994	0.978–1.010	0.44

*Smoking-related cancer (showing ICD-9 code number in brackets): lip (140), tongue (141), mouth and pharynx (143–149), esophagus (150), pancreas (157), respiratory tract (160–163) and urinary tract (188–189).

**The following risk factors (RF) were adjusted for both baseline risk and age: age at baseline, major disease at baseline, systolic blood pressure, random serum glucose, serum uric acid, alcohol use, smoking, marital status, body mass index, and lived in Japan when young.

We used a simple regression model to examine the relation between fasting insulin level and height at HHP Exam 4 with fasting insulin level as the dependent variable (n = 3,458 subjects), adjusting for age at Exam 4. This yielded a regression coefficient of 0.26 (95% CI 0.10–0.42; *P* = 0.001), demonstrating that every 1 cm increase in height corresponded to an increase of 0.26 mIU/L in fasting insulin level.

An analysis of the HHP/HAAS subpopulation described above divided according to median height (161.4 cm) into shorter and taller categories, demonstrated higher prevalence of the *T* allele of the *FOXO3 rs2802292* SNP in the taller subgroup (Table 5A). A phenotype-genotype analysis showed, moreover, that taller baseline height was associated with the *T* allele, *TT* subjects being 1.4 cm taller than *GG*/*GT* subjects (Table 5B).

**Table 5:** Comparison of *FOXO3 rs2802292* genotype with height at Exam 4.

A. Category vs. genotype									
	*GG*	*GT*	*TT*	X^2^	*P*	*GG*/*GT*	*TT*	X^2^	*P*
>161.4 cm*	26	108	159			134	159		
<161.4 cm†	25	138	131	6.4	0.041	163	131	5.5	0.020

B. Height (cm) vs. genotype						
GG	*GT*	*TT*	*P* §	*GG*/*GT*	*TT*	*P*
160.8±5.8	160.5±6.2	161.9±5.9	0.020	160.5±6.2	161.9±5.9	0.005
(n = 51)	(n = 246)	(n = 290)		(n = 297)	(n = 290)	

The subpopulation chosen for this analysis was from participants whose blood sample for genotyping was collected at HHP/HAAS follow-up Exam 4 (in 1991–1993). *The taller subgroup in the contingency table analysis comprised subjects whose height was greater than median height of all subjects (161.4 cm).

† The shorter subgroup comprised subjects whose height was equal to or less than median height of all subjects.

§ *P* shown was result obtained from 1-way ANOVA.

A Cox regression model with time-dependent variables (as explained in the data analysis section of Methods) demonstrated an increase in relative risk of mortality for baseline height as the population aged (Table 6). Prior to age 70 years, relative risk of mortality for each cm increase in baseline height was 0.998, and increased to 1.007, 1.010 and 1.014 for age categories 71–80, 81–95, and >96 years, respectively (*P* = 0.16, 0.088 and 0.016, respectively), compared to RR prior to age 70 years.

**Table 6:** Comparison of RR of mortality for each cm increase in baseline height for different life stages estimated by a Cox regression model.

Life stage (years)	≤70	71–80	81–95	>96
RR for baseline height on mortality	0.998	1.007	1.010	1.014
*P* value compared to those aged ≤70	N/A	0.157	0.088	0.016

RR is shown for each specific life stage. N/A, not applicable.

## Discussion

The present prospective study spanning more than 40 years demonstrates that height is positively associated with increased mortality. Taller study participants not only were at increased risk of death but also tended to have higher fasting insulin levels and lower frequency of the longevity-associated *FOXO3* genotypes *GG* or *GT* of the *rs2802292* SNP. This is among the largest and most detailed of studies to date to have examined this issue and the first to examine the relation of *FOXO3* genotype to human body size, a phenotype of longevity in model organisms.

The relationship between mortality and adult height in humans is complicated. Mortality is related to socio-economic status early and later in life, and other factors such as quality of health care, accessibility to health care facilities and the healthy lifestyle choices of individuals. It is also related to various known and unknown biological processes. Height is both influenced by familial factors and is an indicator of socio-economic status and nutrition during childhood. Currently, the common wisdom in epidemiology is that height in humans in developed nations is a proxy for socio-economic status during early childhood (birth through age 5 years in particular) [27], [28]. Thus taller people are taller due to better medical care and nutrition during early childhood. Therefore, one might expect taller persons to have lower mortality rates.

In support of this hypothesis, several studies have found an inverse association between height and mortality, with the lowest mortality in the tallest individuals within various age groups [1]–[8]. Certain features of these studies are, however, of concern. The age range of participants at baseline was large, e.g., 40–64 years in some [1]–[3] and even greater in an another, consistent with possible cohort bias [4]. In many of the studies a large proportion of participants were in their forties, fifties and sixties at establishment of the cohort, but relatively few studies had large cohorts that were followed to very old age or extinction and still fewer had large numbers of study participants who lived to older age groups (i.e., octogenarians, nonagenarians and centenarians) where we detected a height-mortality effect. Another drawback of these studies was that many used follow-up time, not age, as the time scale in Cox regression models used for their analysis. It is well known that human height tends to decrease in the elderly. In general, all else being equal, we found that older persons at the baseline examination (aged in their 60 s) were shorter than younger persons (aged in their 40 s; see Table 2). The rate of all-cause mortality and CHD mortality both increase substantially in mid to later life. According to the Centers for Disease Control and Prevention mortality data for the US population from 1968 to 1998, the annual death rate of males in America males increases from <1% at ages 45–54 to ≥6% for ages 75–84 years. If follow-up time is used as the time scale, the combination of shorter height in older men and higher mortality in the older age group artificially biases results towards an inverse association between height and mortality. In fact, use of follow-up time as the time scale will not only overestimate the inverse association between height and mortality should an inverse association exist, but may produce an inverse association when this does not exist. This phenomenon could explain why the height-mortality association declined with length of follow-up in the Whitehall Study [3]. The authors of that study found that by 15 years of follow-up, the only appreciable effect of height was on mortality from respiratory disease. As follow-up time increased, the artificial effect produced by the incorrect time-scale decreased. Therefore, to avoid bias, when a Cox proportional hazard regression model is used for evaluation of human mortality, age, instead of follow-up time, should be used as the time scale [29], [30].

Interestingly, in earlier studies of the current cohort of aging men, we noted that taller men had lower prevalence of poor cognitive performance and Alzheimer’s disease suggesting that better nutrition and other favorable early life conditions might provide some later-life health advantages [31]. Stronger grip strength, another phenotype influenced by optimal early life nutrition, also predicts longevity in the HHP cohort and other populations [32]–[34]. Nevertheless, despite the influence of what may have been a healthier start in life, by late life, on average, shorter men outlived their taller counterparts.

Clinically, one might speculate that the slightly reduced overall lifespan of taller individuals on average could be an influence of the well-known increased prevalence of metabolic syndrome and cardiovascular disease in those born small for gestational age who undergo catch-up growth postpartum [14], [15]. Another influence could be from diets high in protein, particularly branched-chain amino acids in meat, that although helping increase linear growth early in life, are associated with reduced lifespan [35], [36]. High protein diets increased risk of cancer and premature death [2], [10], [16], [36]. We found that amongst nonsmoking Japanese American men from the HHP, those whose energy consumption was 15% below average lived longer [17].

The biological reason for an association of shorter stature in humans with longevity is not fully understood. Indeed, the developmental regulation of scaling of body size has been a key question in evolutionary biology for almost a century [37]–[39]. Interestingly, a recent study of *Drosophila* identified *FOXO* as an important genetic regulator of morphological scaling [40]. *FOXO* is a key regulatory gene in the IIS pathway, a nutrient- and energy-sensing biological pathway that is evolutionarily conserved from yeast to humans, and impacts model organism and human longevity [19]. According to this prior work, since nutrition is a primary regulator of body size in animals, and this effect appears to be regulated by FOXO, the evolutionary conservation of the insulin-signaling pathway suggests that *FOXO* may be a proximate evolutionary target of selection for animal morphological scaling. One nutritional cue for scaling for smaller body size could be a lack of calories. This may be a highly conserved stress response that evolved early in life’s history to increase an organism’s chance of surviving adversity [41]. The biological response appears to be altered *FOXO* expression during development, with resultant phenotypic changes including increased insulin sensitivity, smaller body size and longer lifespan [42], [43].

The present study supports this work, finding that the longevity-associated *G* allele of the *FOXO3* SNP *rs2802292* (genotypes *GG* and *GT*) [19] is associated with shorter stature. Since FOXO3 is a transcription factor involved in insulin/IGF-1 signaling and the longevity/short stature-associated *G* allele is associated with lower fasting insulin [19], a possible biological explanation is apparent.

Using the Cox regression model with age as the time scale, stratified by year of birth, effectively reduced bias, and allowed the true relation (estimated RR) of the effect of baseline height on mortality to become apparent. Since our study utilized a genetically homogenous population American men of Japanese ancestry [19], possible confounding from cultural differences were substantially ameliorated, thus reducing bias and strengthening the reliability of the findings.

## Conclusions

The present longitudinal study involving a relatively homogenous population followed for more than 40 years has found height to be positively associated with mortality. *FOXO3* genotype might be a contributing factor to this association. Replication is required in other settings in order to determine whether the present findings can be generalized to other racial groups and populations.

## Supporting Information

Appendix S1 **Use of time-dependent covariates in Cox regression model to estimate relative risks of baseline height for different life stages (age ≤70, 71–80, 81–95, and >95 years).**
(DOCX)
