# Correction: Shorter Men Live Longer: Association of Height with Longevity and *FOXO3* Genotype in American Men of Japanese Ancestry

**DOI:** 10.1371/journal.pone.0105839

**Published:** 2014-08-13

**Authors:** The PLOS ONE Staff

There are several errors in the author affiliations. The first, fourth, and fifth authors are also affiliated with: Research and Development Office, VA Pacific Islands Health Care System, Honolulu, Hawaii. The affiliations should appear as shown here:

Qimei He^1,2,3,4^, Brian J. Morris^1,5^, John S. Grove^1,6^, Helen Petrovitch^1,2,3,4^, Webster Ross^1,2,3,4^, Kamal H. Masaki^1,3^, Beatriz Rodriguez^1,3,6,7^, Randi Chen^1^, Timothy A. Donlon^1,8^, D. Craig Willcox^1,3,9^, Bradley J. Willcox^1,2,3,7^

1 Honolulu Heart Program/Honolulu-Asia Aging Study, Physicians’ Office Tower, Kuakini Medical Center, Honolulu, Hawaii, United States of America

2 Pacific Health Research and Education Institute, Honolulu, Hawaii

3 Department of Geriatric Medicine, John A. Burns School of Medicine, University of Hawaii, Honolulu, Hawaii, United States of America

4 Research and Development Office, VA Pacific Islands Health Care System, Honolulu, Hawaii

5 School of Medical Sciences, University of Sydney, Sydney, New South Wales, Australia

6 Department of Public Health, University of Hawaii at Manoa, Honolulu, Hawaii

7 Instituto Tecnologico de Monterrey, Monterrey, Mexico

8 Department of Research, Kuakini Medical Center, Honolulu, Hawaii, United States of America

9 Department of Human Welfare, Okinawa University, Ginowan, Okinawa, Japan

The following information is missing from the Funding section: This study was supported by Kuakini Medical Center.

In the Materials and Methods section, under the title “Subjects for *FOXO3* Genetic Association Study,” the first sentence should read “Later (in 2007), a subset of 615 subjects who participated in Exam 4 were chosen for a genetic association study of single nucleotide polymorphisms (SNPs) of *FOXO3* and longevity, as reported previously [19].”

In Table 4, the sixth column should be titled “Age and RF Adjusted.” The correct version of Table 4 can be viewed below.

**Table 4:** Relative risk for height (per cm increase) for various conditions using data up to the end of 1999.

Type of mortality	No. of events	Age adjusted			Age and RF adjusted **		
		RR	95% CI	*P* value	RR	95% CI	*P*
Total mortality	5154	1.006	1.002–1.011	0.010	1.005	1.000–1.010	0.076
All cancer	1584	1.013	1.004–1.022	0.004	1.010	1.001–1.019	0.029
• smoking related*	632	1.008	0.995–1.022	0.238	1.002	0.988–1.017	0.74
• not smoking related	952	1.016	1.005–1.028	0.006	1.015	1.003–1.027	0.012
• stomach (ICD 151)	249	1.009	0.987–1.031	0.434	1.010	0.987–1.033	0.41
• colorectal (ICD 153, 154)	209	1.005	0.981–1.029	0.687	1.003	0.978–1.028	0.82
• prostate (ICD 185)	170	1.025	0.998–1.053	0.072	1.023	0.995–1.051	0.10
• hematopoietic (ICD 200-8)	120	1.038	1.006–1.072	0.019	1.041	1.008–1.075	0.015
CHD	757	1.007	0.994–1.020	0.291	1.003	0.991–1.017	0.60
Respiratory disease	389	0.999	0.981–1.016	0.877	1.000	0.982–1.018	0.99
Stroke	493	0.994	0.979–1.010	0.484	0.994	0.978–1.010	0.44

*Smoking-related cancer (showing ICD-9 code number in brackets): lip (140), tongue (141), mouth and pharynx (143–149), esophagus (150), pancreas (157), respiratory tract (160–163) and urinary tract (188–189).

**The following risk factors (RF) were adjusted for both baseline risk and age: age at baseline, major disease at baseline, systolic blood pressure, random serum glucose, serum uric acid, alcohol use, smoking, marital status, body mass index, and lived in Japan when young.
