# Supplementary material for: Shorter Men Live Longer: Association of Height with Longevity and FOXO3 Genotype in American Men of Japanese Ancestry
Source: PLoS One. 2014 May 7;9(5):e94385. doi: 10.1371/journal.pone.0094385 (PMC4013008; doi:10.1371/journal.pone.0094385)
Supplement: Appendix S1 — Use of time-dependent covariates in Cox regression model to estimate relative risks of baseline height for different life stages (age ≤70, 71–80, 81–95, and >95 years). (DOCX) [file pone.0094385.s001.docx]

**Supporting Information**

**Appendix S1. Use of time-dependent covariates in Cox regression model to estimate relative risks of baseline height for different life stages (age ≤ 70, 71–80, 81–95, and >95 years)**

First, index variables for each of the life stages are created: A1 = 1; if age ≤ 70, or else A1 = 0; A2 = 1, if age between 70 and 80 years, else A2=0; A3=1, if age between 80 and 95 years, else A3=0; A4=1 if age > 95 years, else A4=0. Then let:

H1 = A1*baseline height,

H2 = A2*baseline height,

H3 = A3*baseline height,

H4 = A4*baseline height;

This gives 4 variables, H1, H2, H3, H4; each equals baseline height inside corresponding life stage, and equals 0 outside the life stage.

Putting these 4 variables (H1, H2, H3, H4) in one Cox regression model, one can estimate the regression coefficient beta_i for each of thse, where exp (beta_i) is the relative risk for mortality for each of the variables Hi and is the relative risk of baseline height for mortality at the corresponding life stage. Please note that the sum of these variables (H1, H2, H3, H4) will be equal to the original height variable.

More specifically, in this model, all participants were used to estimate the relative risk on baseline height for mortality in the first life stage (age < 70 years), all participants who had died after the age of 70 years were used to estimate the relative risk of baseline height for mortality in the second life stage (ages 70–80 years), all participants who died after age 80 were used to estimate the relative risk of baseline height for mortality in the third life stage (80–95 years), and all participants who died after age 95 were used to estimate the relative risk of baseline height for mortality in the fourth life stage (ages >95 years). Separated models can be used to estimate the relative risk of baseline height on mortality in different life stages. However, in these models, the effects of confounding variables will be different between different models, and there is no ideal mechanism to compare the relative risks of baseline height at different life stages.

**Below is the SAS program code to analyze this type of data** (one can add the confounding variables in the model in a similar manner as one would do for models without time-dependent variables, and where age is the time scale):

Proc PHREG;

Model age*tranc(0)=H1 H2 H3 H4;

If age < 70 then a1=1; or else a1=0; H1=height*a1;

if 70≤ age and age <80 then a2=1; else a2=0; H2=height*a2;

if 80≤ age and age 95 then a3=1; or else a3=0; H3=height*a3;

if age ≥95 then a4=1; or else a4=0; H4=height*a4;

run
